# Ecosystem service value in the context of urbanization: Comparison among economic-social regions of Vietnam

**DOI:** 10.1016/j.heliyon.2024.e39878

**Published:** 2024-10-26

**Authors:** Pham Lan Huong, Nguyen Tran Tuan

**Affiliations:** Faculty of Real Estate and Resources Economics, National Economics University, 207 Giai Phong, Hanoi, 113068, Viet Nam

**Keywords:** Land use change, Remote sensing, Spatiotemporal, Quantify ESV, Sustainable development

## Abstract

Urbanization is one of the important features of socio-economic development and it has a major impact on the value of ecosystem services (ESV). This study aims to quantify ESV for the entire country of Vietnam with the research unit being 6 socio-economic regions. This can be considered the first study in Vietnam to quantify the ESV for the entire country. The study calculated the annual urban land growth index (AI) and the annual urban land expansion rate (AER) to determine the urbanization status in the six study areas. The study collected input data from JAXA for four years: 1990, 2000, 2010, and 2020. Research results show that urban land area tended to increase and forest land area decreased sharply. The AI and AER indicated that the period 2000–2010 was a period of rapid growth in Vietnam. Meanwhile, the ESV for all of Vietnam increased year by year. The period 2000–2010 witnessed a growth in ESV about 2.35 times higher than the period 1990–2000, and about 0.1 % lower than the period 2010–2020. Overall, MKD was the region showing strong growth in ESV from 1990 to 2020. Compared to that, NMR was the region with the largest decline in the 20 years from 1990 to 2010, and CHR in the past 10 years. This research contributes to enriching reference sources for Vietnamese and world researchers to have a broader view of ESV as well as the annual urban growth rate in Vietnam from 1990 to 2020.

## 1 Introduction

The sustainability of ecosystems is determined by understanding the benefits they provide to human society and ecosystem services [1]. These services are defined by their ability to provide ecological and cultural resources and life support services [2,3]. These services can be identified through monetary, aesthetic, and spiritual value in human development [4]. The product can be exchanged or paid for on the market. Quantifying the values of ecosystem services is becoming increasingly popular in the current context of resource depletion. It aims to leverage understanding of environmental controls and anthropogenic impacts in space to ensure balanced development goals for the territory [[5], [6], [7]]. However, disturbances that change environmental conditions come largely from land use and surface cover [8,9], which are areas where urbanization is intensifying and leading to environmental degradation [10]. Therefore, the method of determining ESV from parameters on the land cover map is considered an indirect approach to assessing anthropogenic impacts on the ecosystem [[11], [12], [13]].

Many studies have used remote sensing image data to extract information on urbanization processes and assess environmental consequences using ecosystem services [[14], [15], [16], [17]]. Using a variety of environmental indicators, it also aims to ascertain how urbanization affects the environment [18,19]. The quantity and stability of satellite data have certain limits, but overall, there have been more studies on ecological services. Customized assessment techniques can account for unique local circumstances and assess the effects of urbanization using a variety of spatial variables [20,21]. The possible advantages or disadvantages of urbanization can then be ascertained by observing changes in the values of ecosystem services [22,23].

The use and exploitation of ecosystem services have added significant value to the Vietnamese economy, especially in the tourism, health, forestry, fishery, and agriculture sectors [24]. As per the 2021 Vietnam Statistical Yearbook, the sectors of agriculture, forestry, and fisheries provide a substantial contribution to the country's GDP. From 2010 to 2020, the export value of these sectors increased from USD 19 billion to USD 41.25 billion, accounting for 14.6 % of the country's total export revenue [25]. When the goods from forestry, wetland, marine ecosystem services, and agriculture are taken into consideration, the total domestic product of these industries in 2018 at current prices is VND 1,221,952 billion (USD 48 billion), or 22.04 % of the overall output [26]. This demonstrates the direct usage values (or economic significance and worth) of the forest, wetland, and marine ecosystem services in Vietnam. Furthermore, the choice, non-use, and indirect use of ESV have not yet been thoroughly assessed and tallied. In addition, there is lack of research on the valuation of ecosystem services in the context of urbanization in Vietnam. There are two studies that have addressed this issue: Pham et al. (2019) [27] and Pham and Lin (2023) [28]. However, these two studies only focused on one specific city in Vietnam, so they could not cover all the ecosystem service values in this country. Therefore, this study is the first in Vietnam to quantify ESV for the entire country and compare the ESV cross six socio-economic regions. The focus of the study is on urbanization and its consequences for ecosystem values at the national level. To fulfill the study's objectives, this article provides answers to the following research questions.

- How does the process of expanding urban land in Vietnam take place in 6 economic regions from 1990 to 2020?
- How has the value of ecosystem services in six economic regions changed from 1990 to 2020?

To answer the two research questions mentioned above, in the next section, this study reviews some documents on the relationship between urbanization and ESV. Then, in Section 3, the research methods and research site overview are listed. The next section will include the research results and a discussion of these findings. Finally, there is a conclusion that answers the research questions and points out the importance and limitations of the study. Besides, this research contributes to enriching reference sources for Vietnamese and world researchers to have a broader view of ESV and the annual urban growth rate in Vietnam from 1990 to 2020.

## 2 Overview of some studies on the relationship between urbanization and ecosystem service values

Many studies have examined the relationship between urbanization and ecosystem service values [29]. Researchers have utilized numerous study models to ascertain spatial and temporal correlations in this discipline. Three models, telecoupling [30,31], decoupling [32], and coupling coordination [33,34], are used to access the influence of urbanization on ESV. Various techniques, including segmented linear regression models [35] and spatially weighted regression models [36], are used to assess the influence of urbanization on ESV. Out of the models mentioned, the land-use change assessment model is widely used due to advances in geographic information systems and remote sensing technologies [37,38]. These models’ outcomes are divergent and even exhibit inconsistencies. Urbanization can improve ecological services by optimizing industrial structures and strengthening urban management [39], but can also lead to a decrease in biodiversity, deterioration of land-use systems, landscape fragmentation, reduction of human benefits, and pollution emissions [40]. Recent research has focused on examining the effects of land-use changes resulting from construction or measuring the correlation between urbanization and housing using urbanization indices.

Urbanization can have either a detrimental or beneficial impact on environmental services [41]. Urbanization alters the composition and functioning of ecosystems, impacting ecosystem services [42]. The process of urbanization, which includes socio-economic development, urban space expansion, and lifestyle changes, has the potential to degrade ecosystem services [43]. Alternatively, the growth of urban populations and industrialization will result in a higher need for and utilization of ecosystem services [44]. Prior research has demonstrated that urbanization impacts the ecosystem services provided by each specific area [45]. Nevertheless, this impact extends beyond specific ecosystems, even affecting ecosystems in adjacent regions [46]. The impact of excellent urbanization performance on nearby urban areas is evident, thus affecting neighboring ecosystems as well.

Recent research has concentrated on examining the effects of changes in land use resulting from the growth of construction areas or measuring the correlation between urbanization and habitat using urbanization indices [47]. Existing research indicates that alterations in land use resulting from human activities have adverse effects on ecosystem services [48]. For instance, the expansion of traffic routes can invade natural areas and worsen the fragmentation of landscapes [49]. Land use change can have a significant impact on several hydrological processes, including rainfall retention, evapotranspiration, and infiltration. These processes can be influenced by alterations in subsurface characteristics [50]. Moreover, the correlation between urbanization and ecosystem service values may vary depending on the choice of urbanization indices used to assess different facets of urbanization [51]. Multiple research findings emphasize the significance of assessing the diverse effects of urbanization on environmental services from a comprehensive urbanization standpoint [52,53].

Additionally, different natural environmental conditions and levels of socio-economic development in different regions lead to spatial heterogeneity in urbanization and ESV. Existing studies consider urbanization's growth scale in terms of population concentration [54], land expansion [55], and economic development [56]. In some cases, the results are contradictory due to different urban contexts. Many studies have shown a gradual decrease in ecosystem service values in urbanized areas due to the negative impacts of rapid land use conversion [57], while urban expansion increases ecosystem service values in the Yellow River Basin, China, due to the construction of ecotourism projects that have increased the area of forests and grasslands [58]. Additionally, different types of land have different effects on ecosystem service values, and ecosystem service values at different levels respond in different ways to the same factor [59]. Using different urbanization indices to measure different aspects of urbanization may alter the relationship between urbanization and ecosystem service supply. When measuring urbanization in terms of land use cover, there is a negative correlation between ecosystem service supply and it, but an inverted U-shaped relationship emerges when measuring it in terms of population and economy [60]. This observation reveals that urbanization's influence on ecosystem services evolves over time, as does the calculation of urbanization indices [61].

The literature review above concludes that studies using different methods of measuring the urbanization index may yield different results. Besides, assessment methods based on land use changes over space and time are widely applied to illustrate the relationship between urbanization and ESV. Moreover, it is crucial to conduct a comprehensive study of this relationship across a broad geographic scope. Therefore, this study calculates urbanization levels through two annual urban land growth indexes (AI) and an annual urban land expansion rate (AER) and quantifies the ESV of six socio-economic regions in Vietnam. However, there are many factors affecting ecosystem value, so this study only evaluates the main determinants of ecosystem value in Vietnam instead of quantifying each factor.

## 3 Methodology

### 3.1 The location of research

Vietnam is located in the center of Southeast Asia, with diverse terrain including mountains, plains, coasts, and continental shelves. Vietnam's varied topography is the result of many years of topographical and geological change under a monsoon-driven climate that is hot, muggy, and heavily worn. Vietnam faces the sea on its three eastern, southern, and western sides, with a 3260-km coastline. Stretching east and southeast, the area of the East Sea under Vietnamese sovereignty is encircled by big and small islands, archipelagos, and the continental shelf. Vietnam, which is part of the inner tropical belt, experiences year-round high temperatures and humidity. Because of mainland China's influence, the climate in the north is predominantly continental. The East Sea greatly influences the humid tropical monsoon climate on the mainland. Vietnam's humid tropical monsoon climate is not uniform throughout the country; instead, it is divided into several distinct climate zones. Vietnam's climate varies from low to high, north to south, and east to west on a seasonal and regional basis. Because of the northeast monsoon's great influence, Vietnam's average temperature is lower than that of many other Asian countries at the same latitude.

**Fig. 1:**
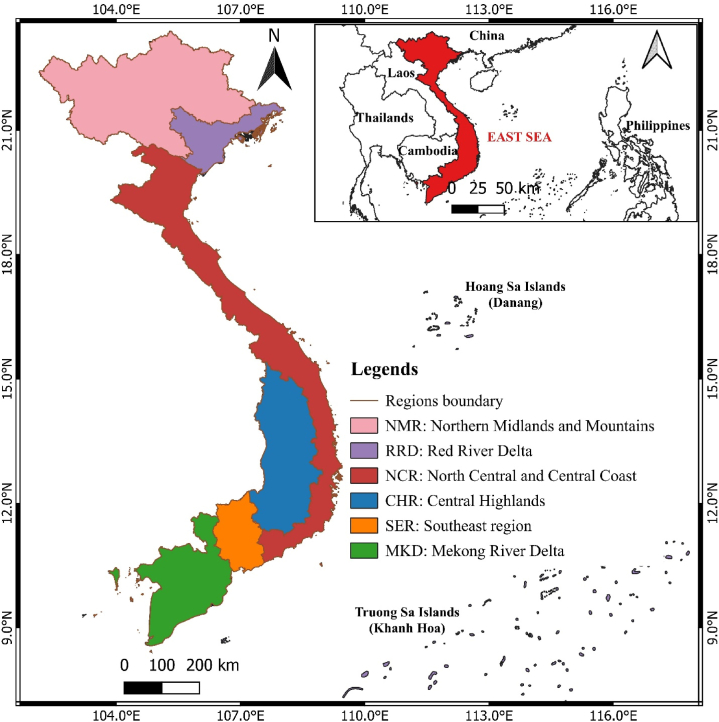
The location and division of Vietnam.

**Source:** authors' compilation, 2024.

Vietnam is acknowledged as having one of the highest levels of biodiversity in the world, with a wide range of species, ecosystems, and uncommon, valuable, and indigenous genetic resources. The ecosystems with the highest levels of biological productivity and biodiversity are forest, wetland, marine, and coastal habitats. These ecosystems also happen to be the primary ones that offer humans the majority of their essential functions. The current decline in biodiversity resources and the degradation of ecosystems are primarily due to Vietnam's robust socioeconomic development. Moreover, Vietnam is divided into six socioeconomic regions in terms of administrative boundaries (Fig. 1). Depending on the natural and economic conditions of each region, the state determines separate development strategies for each region.

### 3.2 Research methods and data analysis

This study aims to quantify ESV in Vietnam in terms of urbanization. So, the first task of the research is to determine urbanization indicators in Vietnam and socio-economic regions in this country. There are two indices: the annual urban land growth index (AI) and the annual urban land expansion rate (AER). The AI index compares urban regions at two distinct times to provide a relative evaluation of urban change (formula 1), whereas the AER index shows the total rate of change throughout that time (formula 2). These two indexes are determined using the formula:

(1) $$AI=\frac{A_{t2}-A_{t1}}{A_{t1}}\;x\;100\%$$

(2) AER=[(At2At1)1T−1]x100%

$A_{t1}$ and $A_{t2}$ are the area of urban land at time t1 and t2, respectively.

T is the time period from t1 to t2.

Quantifying ESV is measured by formula (3):

(3) $$ESV=\sum \left(A_{i}\;x\;{VC}_{i}\right)$$

$A_{i}$ is the area of land i; ${VC}_{i}$ is the value coefficient.

${VC}_{i}$ is determined based on the research of Pham and Lin (2023) [28]. These are specifically expressed in Table 1. The global ESV coefficients from Costanza et al. (2014) [62] determined these values. This is one of the most comprehensive sets of ESVs compiled from numerous studies around the world. To be more accurate, Pham and Lin (2023) [28] used an average of 25 % green space for high-density and 75 % green space for low-density urban pixels. They arrived at this conclusion after looking at satellite images and learning about how Vietnamese cities work. This adjustment allows for a more accurate calculation of urban land because it reflects its value in terms of its green ratio. The ESV coefficients of each land use type in Vietnam were adjusted to 2020 USD values using the consumer price index.

**Table 1:** Value coefficient of ESV.

Class	High-developed areas	Low-developed areas	Crops	Grass-land	Scrub	Forests	Water	Aquaculture	Barren land
**VC ($ha**^**−**^**^1^ yr**^**−**^**^1^)**	2091	6273	6904	5166	4713	6672	15,515	31,844	0

In addition, it is necessary to ascertain both the extent of urban land and the dimensions of land strata. The study obtained data from JAXA spanning a four-year period, specifically in 1990, 2000, 2010, and 2020. The overall accuracy rates for these years were 78 %, 79 %, 82 %, and 86 %, respectively. The land cover products in Vietnam are classified into 18 distinct land use types using techniques like random-forest-based algorithms and geospatial satellite image data such as Landsat, Sentinel-1, and Sentinel-2. To make computation easier, the authors consolidated land layers with comparable characteristics and established nine land layers, as shown in Table 1. Furthermore, while assessing the value of the AI and AER indices, the high- and low-developed land classes are merged into the urban land category to simplify the computation process. The data is subsequently analyzed using QGIS 3.36.1 software to ascertain the extent of land strata throughout Vietnam, as well as in socio-economic zones.

## 4 Results

### 4.1 Land use statistics from 1990 to 2020

**Fig. 2:**
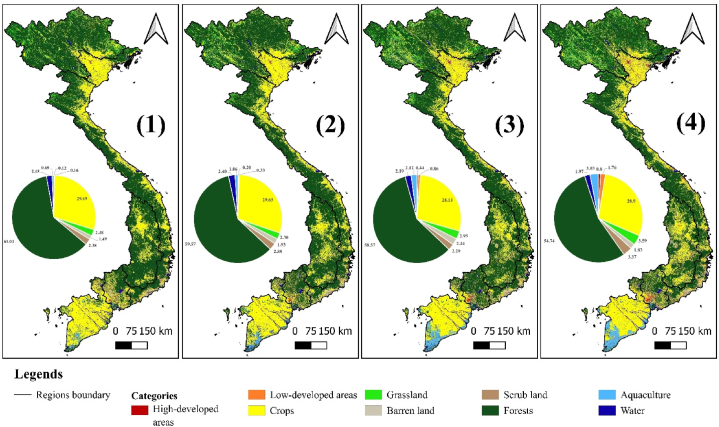
Lulc in Vietnam from 1990 to 2020. **Note:** (1): LULC in Vietnam in 1990; (2): in 2000; (3): in 2010; (4): in 2020.

**Source:** authors' compilation, 2024.

From 1990 to 2020, Vietnam's land layers showed a significant change in distribution and ratio (Fig. 2). Statistical data on land areas of the regions can be seen in Appendix A, Appendix A. The area of forest land was concentrated in the west, while cropland was scattered in the east. The cropland group was larger in RRD and MKD regions, while urban land (including high-developed and low-developed areas) was larger in the RRD and SER regions. These are also known as two large rice granaries in Vietnam, with the addition of two large rivers, the Red and Mekong Rivers. However, in the CHR region, the area of cropland was larger than before. Aquaculture areas were also more prominent in the MKD region.

The area of forest land decreased significantly after 30 years, with the largest decrease occurring in the last decade (2010–2020). The area of this land group remained almost unchanged from 2000 to 2010. However, the urban land and aquaculture groups experienced growth, with aquaculture increasing the most significantly. The urban land group showed growth of 1.5 % in the low-developed areas group and 0.7 % in the high-developed areas group. In addition to these two trends, the remaining land groups showed up-and-down fluctuations over each period. Over the years, only the cropland group saw almost no change. This was also the second-largest land group in Vietnam after forests.

### 4.2 Comparing annual urban growth results

Table 2 shows the urban land growth index and the annual urban land expansion index in six socio-economic regions and the whole of Vietnam. The period 2000–2010 was the strongest development period in Vietnam, with the largest annual growth of urban land in the CHR region. The period 2010–2020 was higher than the period 1990–2000 in AI and AER, but this difference was not significant. Among the six economic regions, MKD was the region with the lowest growth, but MKD was the region with the fastest growth during the transition period from 1990 to 2000 to 2000–2010. Similar to the overall growth trend of Vietnam in both indexes, economic regions tended to increase in the middle period and decrease their level of development in the past 10 years. However, the RRD area showed both annual urban expansion and a downward trend in annual urban growth during each period.

**Table 2:** AI and AER.

Percent	AI			AER		
	1990–2000	2000–2010	2010–2020	1990–2000	2000–2010	2010–2020
NMR	1	1.96	1.59	7.16	11.46	9.99
RRD	1.13	1.05	0.74	7.86	7.47	5.72
NCR	1.07	1.54	1.14	7.57	9.76	7.89
CHR	2.16	2.53	2.24	12.18	13.43	12.47
SER	0.86	1.19	0.64	6.42	8.13	5.1
MKD	0.52	2.06	0.96	4.28	11.84	6.97
**Vietnam**	**0.92**	**1.46**	**0.96**	**6.72**	**9.42**	**6.96**

### 4.3 Comparing the ESV

Table 3 shows the results of changes in the quantitative ESV of land layers in regions in Vietnam from 1990 to 2020. The ecosystem services value in Vietnam increased year by year, with the forest land group contributing the most to the value. The period 2000–2010 witnessed a growth in ESV that was about 2.35 times higher than the period 1990–2000 and about 0.1 % lower than the period 2010–2020. Among land groups, the ecosystem service value of forests decreased every year, with the contribution of less developed areas being the lowest. In particular, the period 2010–2020 witnessed the sharpest decrease in the ecosystem service value of forest land, with over 8800 million US$/ha/year (Appendix 3), equivalent to about 6.5 %. Crops, with varying ESV, were the second-largest contributing land group. From 1990 to 2000, this value showed a slight increase of 0.47 % before a sharp decrease of 5.05 % in the period 2000–2010. Then, at the final stage, the value in the crop land layer increased to 2.71 %. Although the urban land group had the lowest contribution in terms of ESV, it had the greatest growth. In the urban land group, low-developed areas had greater growth than high-developed areas. The low-developed area land group was the only one to show growth of over 100 % in all three research periods.

**Table 3:** The change of ESV.

%		High-developed areas	Low-developed areas	Crops	Grass-land	Scrub	Forests	Water	Aquaculture	Total
**1990–2000**	NMR	55.06	141.13	10.26	−4.41	−60.85	−3.01	10.60	−6.74	−1.18
	RRD	68.08	173.02	0.31	−48.25	134.48	−3.26	0.90	113.31	1.42
	NCR	71.70	177.31	−3.03	−20.01	15.50	−0.44	11.44	27.69	−0.11
	CHR	178.73	332.69	20.12	−91.20	61.10	−7.96	54.38	84.81	−0.55
	SER	53.50	113.32	−26.12	159.42	−5.69	16.50	13.31	−22.32	1.35
	MKD	−3.98	55.98	−3.43	299.92	7.19	−6.50	13.93	58.73	9.16
	**Total**	**65.21**	**111.63**	**0.47**	**−7.31**	**8.38**	**−2.41**	**11.47**	**54.06**	**1.17**
**2000–2010**	NMR	230.78	175.06	3.66	32.22	−59.59	−4.41	2.19	140.34	−0.99
	RRD	128.93	86.23	−8.34	−13.23	−32.45	5.50	3.94	61.39	0.41
	NCR	83.87	238.72	−9.30	33.78	1.45	1.48	3.64	39.76	0.07
	CHR	189.26	381.90	5.80	900.53	23.54	−4.69	73.48	61.71	−0.49
	SER	110.91	123.08	−34.17	−3.94	−40.24	15.63	−3.87	149.63	1.20
	MKD	286.42	202.87	−5.62	−29.40	−30.54	−22.98	−30.37	105.76	17.61
	**Total**	**123.75**	**159.06**	**−5.05**	**28.61**	**−11.00**	**−1.68**	**−8.78**	**99.96**	**2.76**
**2010–2020**	NMR	127.99	181.28	−7.15	29.02	−5.33	−1.11	13.15	304.47	1.49
	RRD	81.18	67.72	−10.01	−16.58	31.35	0.31	−18.23	187.45	4.92
	NCR	98.17	123.93	4.07	−11.16	36.78	−3.91	−4.59	115.97	1.65
	CHR	160.80	300.62	27.23	324.09	77.76	−17.03	16.31	579.73	−0.85
	SER	26.30	85.83	62.04	−47.37	41.10	−22.10	2.73	77.89	2.19
	MKD	55.43	98.36	−5.23	−44.68	12.66	−11.48	−27.84	28.34	8.46
	**Total**	**82.04**	**103.15**	**2.71**	**21.66**	**47.05**	**−6.53**	**−9.80**	**43.05**	**2.81**

If we compare the change in ESV between regions, it is simple to see that the region with the greatest decrease in forest area shows a negative value. For example, in the period 1990–2000, the ESV of forests in the three regions NMR, NCR, and CHR decreased by 3.01 %, 0.44 %, and 7.96 %, respectively. The ESV of these three regions decreased by 1.18 %, 0.11 %, and 0.55 %, respectively. The MKD region experienced a decrease in the value of forest ecosystem services, while its ecosystem service value increased due to the aquaculture land group's strong growth. This trend is repeated for regions in the next two periods. Overall, MKD showed strong growth in ecosystem service value from 1990 to 2020. Compared to that, NMR was the region with the largest decline in the 20 years from 1990 to 2010, and CHR in the past 10 years.

Table 4 shows the value of each ecosystem service for the whole of Vietnam from 1990 to 2020. Overall, climate regulation, genetic resources, food, and recreation were the main contributors to the total value of ecosystem services in Vietnam. However, the total value of services in the Provisioning Services category was on a downward trend, while the total value of services in the other two categories was on an upward trend. The fact that the majority of the first group's independent ecosystem services rely on forest and cultivated land could explain its decline. In addition, corresponding to the period of the fastest urban development in Vietnam, the period 2000–2010 also witnessed the largest decline in the total value of ecosystem services in the Provisioning Services category, with over 2000 million US/ha/year. Meanwhile, the total increase in the value of individual ecosystem services in the remaining two categories was similar between the two periods 2000–2010 and 2010–2020. The tables in this article's supplementary document, from S1 to S4, show the service value of each ecosystem in the regions over time.

**Table 4:** Value of the individual ecosystem service in Vietnam.

Million US/ha/year		1990	2000	2010	2020
Provisioning Services	Food	36,512.38	36,564.42	35,557.42	36,674.77
	Material	5352.03	5407.00	5471.65	5702.41
	Water	7769.55	8042.14	7798.11	7966.84
	Genetic Resources	54,771.45	53,833.79	52,843.89	51,272.61
	**Total**	**104,405.42**	**103,847.35**	**101,671.06**	**101,616.63**
Regulation and Maintenance	Gas Regulation	330.85	322.85	319.65	304.08
	Climate Regulation	59,804.86	58,731.34	57,913.74	55,518.55
	Disturbance	2636.72	3073.57	4401.75	5472.29
	Water Regulation	8822.39	10,509.53	12,377.83	13,871.73
	Erosion Control	11,120.66	11,335.41	12,308.12	12,869.44
	Soil Formation	7120.19	7144.39	6794.96	6953.88
	Nutrient Cycling	658.37	935.62	1705.68	2402.32
	Waste Treatment	10,121.85	10,652.98	11,641.75	12,747.87
	Pollination	1104.54	1085.03	1066.89	1038.62
	Biological Control	1201.11	1359.57	1753.05	2203.99
	Habitat Refugia	3667.13	3993.50	5361.82	6881.68
	**Total**	**106,588.68**	**109,143.79**	**115,645.25**	**120,264.43**
Cultural Services	Recreation	27,808.19	28,305.12	29,725.79	31,338.47
	Cultural	809.50	1113.75	2066.59	2890.81
	**Total**	**28,617.69**	**29,418.87**	**31,792.38**	**34,229.28**

## 5 Discussion

In recent decades, urbanization has been taking place strongly around the world, particularly in developing countries. This process helps improve people's quality of life, create job opportunities, and promote economic development [63,64]. In addition, urbanization has had a significant impact on the environment and ecosystems of developing countries, particularly Vietnam [65,66]. This study quantified the ESV for the entire country of Vietnam as well as compared changes in this value from 1990 to 2020 in the context of urbanization.

The country's urban environment is divided into two urban hubs: Hanoi and surrounding provinces, which form the northern urban hub, and the southern urban hub, which includes Ho Chi Minh City [67]. This is clearly demonstrated in Fig. 2's spatial land use distribution map. Hanoi and the surrounding provinces make up the northern urban pole. It extended its influence on the NMR, RRD, and northern NCR regions. In the meantime, the influence extended to CHR, SER, MKD, and the southern NCR region, with Ho Chi Minh City serving as the focal point of the southern urban pole. These metropolitan areas serve as the nation's economic engines. Together, these urban centers account for 80 % of all industrial and service employment in Vietnam and have emerged as the country's most dynamic and geographically accessible places [68]. However, this concentration leads to an uneven distribution of urbanization across the six socio-economic regions. This data is also clearly seen through urban land use statistics and urban land use rates in regions in Vietnam in Appendix A, Appendix A.

According to Tuan and Hegedűs (2022) [69], urbanization in Vietnam is linked to the movement of workers and their families from rural to urban areas. People moving from the agricultural to industrial sectors in metropolitan areas is another example of this change. In particular, Vietnam's urbanization rate increased rapidly after the 1986 economic reform period. However, the pace of urbanization in Vietnam has slowed down somewhat since 2010–2020. This is similar to the research results shown in Table 2. This slowdown also reflects a slower rate of migration from rural to urban areas. From 1989 to 1999, there was a dramatic growth in the migrant population, which jumped from 2.4 million to 4.5 million, representing 4.5 % and 6.5 % of the total population aged 5 and older, respectively [70]. As we enter the decade 1999–2009, 6.7 million people migrated in 2009 alone, driven by strong economic development, the explosion of industrial parks, and the structural shift in the economy away from agriculture and towards industry and services [71]. At the time, this migratory population accounted for 8.5 % of Vietnam's total population of 5 and up. In the ten years between 2009 and 2019, the disparity in economic opportunities between urban and rural areas was significantly reduced thanks to localized programs and socio-economic projects [72]. As a result, migration rates and overall numbers have dropped over this period. According to Huong (2023) [73], out of the total population of 5 years and older, 6.4 million are migrants, making up 7.3 % of the country's population in the 2019 Population and Housing Census.

In addition to the positive impacts of developing a young workforce and focusing on high-quality human resources, population growth also increases infrastructure pressure. This is one of Vietnam's challenges with urban management. The formation of most urban areas in Vietnam occurred at a young age, and subsequent expansion and uneven development planning led to a lack of consistency and diminished resistance to climate change [74]. There is still a severe shortage of trees, lakes, and water sources in Vietnam. High temperatures and flooding during the rainy season disrupt people's lives, affecting air quality [75]. Reality also shows that it is undeniable that migrant workers are a human resource that plays a major role in supplementing the labor force, promoting the development of diverse economic sectors and industries, and the overall growth of urban areas. However, rapid migration and urbanization not only put pressure on urban infrastructure, but also have a significant impact on the environment [76]. For example, the appearance of various artificial light sources causes disturbances to the biological rhythms of living organisms, as well as imbalances in the ecosystem [77]. Light causes green plants to have a disorder in the photosynthesis mechanism and tend to lose leaves, increasing the amount of CO_2_. Artificial light emitted from urban areas attracts millions of insects, depriving birds of food and disrupting the natural food chain [78].

The above comments show that Vietnam's urbanization process continues, exhibiting signs of unsustainable development. This is evidenced by the fact that, despite an increase in the overall value of Vietnam's ecosystem services, the ESV declined primarily in two land groups: forests and water. These are also the two land groups responsible for the majority of the ecosystem's functions. This situation is even more clearly seen in the NMR, NCR, and CHR regions. These are the three regions that account for most of the forest land area across the country. Additionally, the pressure from rapid population growth, economic expansion, and the increasing demand for ecosystem services is leading to the overexploitation of biodiversity resources. This problem becomes apparent when the forest area decreases over time. For the three years 2016–2018, the average annual damage to forest area was 2430 ha. From 2010 to 2017, the area of natural forests tended to decrease, and the area of planted forests increased [26]. However, planted forests are often pure species, so they are much less diverse than natural forests. Increased coastal water pollution, impacts on marine habitats, and human life are all results of socioeconomic development in coastal locations [79]. As a result, river, stream, and lake ecosystems are degraded, and biodiversity levels decline. These facts are similar to the research results shown in Table 3.

Based on the above observations and analyses, the authors recommend that policymakers reconsider ecosystem-based approaches in their future urban planning efforts. Urban planning should respond to people's needs and protect their well-being rather than expand urban areas in the direction of administrative boundary consolidation, as is currently the case. This necessitates not only integrating urban green spaces into the development process but also enhancing biodiversity and connectivity of existing infrastructure. Additionally, policymakers should link urban land use planning to space, land use zoning, and natural ecosystems. Two important components, land use zoning for urban land and promulgating regulations for each land use purpose, form the foundation of urban land use planning. By effectively combining these two issues, we can enhance the quality of urban areas in a contemporary manner and attain the objective of sustainable urban development. Accordingly, land use zoning aims to demarcate boundaries and arrange land use space into three areas, including strictly managed areas, restricted areas, and areas with converted land use purposes. In addition to zoning, it is necessary to study and determine criteria and norms for important land purposes associated with space, land use zoning, and natural ecosystems for residential land and urban land in the process of planning and urban land use plans. This will be an important basis for accurately calculating the needs, conditions, and development orientations. From there, the government should set specific norms for residential land at each planning level, enhancing the quality of land use planning and fulfilling the demands of sustainable development.

## 6 Conclusions

Urbanization is an important feature of socio-economic development, and it has a significant impact on the value of ecosystem services. Taking six socio-economic regions in Vietnam as the research unit, this paper analyzes the land use transition process in six socio-economic regions of Vietnam from 1990 to 2020, focusing on urban growth and ecosystem service value in the context of urbanization. In the research area and time period, urban land area tended to increase while forest land area decreased sharply. The period 2000–2010 witnessed rapid growth in Vietnam, with the RRD area showing both annual urban expansion and a decreasing annual urban growth trend.

Meanwhile, the ESV for all of Vietnam increased year by year. The period 2000–2010 witnessed a growth in ESV that was about 2.35 times higher than the period 1990–2000 and about 0.1 % lower than the period 2010–2020. Among land groups, the ecosystem service value of forests contributed the greatest value in Vietnam, but this contribution value witnessed a gradual decrease each year. In particular, the period 2010–2020 witnessed the sharpest decrease in the ecosystem service value of forest land, with over 8800 million US$/ha/year, equivalent to about 6.5 %. If we compare the change in ecosystem service value between regions, it is straightforward to see that the region with the greatest decrease in forest area shows a negative value. However, overall, MKD was the region showing strong growth in ecosystem service value from 1990 to 2020. Compared to that, NMR was the region with the largest decline in the 20 years from 1990 to 2010, and CHR in the past 10 years.

The above conclusions have answered the two research questions raised, and they also show the importance of this research. This study is the first in Vietnam to quantify the value of ecosystem services for the six socio-economic regions over a long period, providing a basis for policymakers and planners to understand the interaction between ecosystem services and socio-economic development. However, the study has limitations, such as the accuracy of land use map data and the value coefficient of ecosystem service, which may lead to inaccurate assessments of multitemporal changes. Future research should use field survey data and detailed land classification to overcome these limitations. In addition, future studies should also identify different value coefficients between geographical regions to more clearly reflect the reality of ecosystem values in these regions.

## CRediT authorship contribution statement

**Pham Lan Huong:** Writing – review & editing, Visualization, Supervision, Funding acquisition, Conceptualization. **Nguyen Tran Tuan:** Writing – original draft, Validation, Software, Resources, Project administration, Methodology, Investigation, Formal analysis, Data curation, Conceptualization.

## Data availability

Data included in article/supplementary material is referenced in the article.

## Use of AI tools declaration

The authors declare they have not used Artificial Intelligence (AI) tools in the creation of this article.

## Funding

This research received no specific grant from any funding agency in the public, commercial, or not-for-profit sectors.

## Declaration of competing interest

The authors declare that they have no known competing financial interests or personal relationships that could have appeared to influence the work reported in this paper.

## Footnotes

Appendix A Supplementary data to this article can be found online at https://doi.org/10.1016/j.heliyon.2024.e39878.
