# Supplementary material for: Ecosystem service value in the context of urbanization: Comparison among economic-social regions of Vietnam
Source: Heliyon. 2024 Oct 26;10(21):e39878. doi: 10.1016/j.heliyon.2024.e39878 (PMC11558626; doi:10.1016/j.heliyon.2024.e39878)
Supplement: Multimedia component 1 [file mmc1.docx]

**Table S1.** Value of the individual ecosystem service for each region in 1990

| **Million US/ha/year** | | **1990** | | | | | | |
| --- | --- | --- | --- | --- | --- | --- | --- | --- |
|  |  | **NMR** | **RRD** | **NCR** | **CHR** | **SER** | **MKD** | **Total** |
| Provisioning Services | Food | 6,950.24 | 4,311.39 | 8,902.75 | 4,497.89 | 1,978.29 | 9,871.82 | **36,512.38** |
|  | Material | 1,263.80 | 453.63 | 1,427.08 | 789.22 | 314.81 | 1,103.49 | **5,352.03** |
|  | Water | 1,191.37 | 1,015.35 | 1,824.69 | 776.03 | 558.01 | 2,404.11 | **7,769.55** |
|  | Genetic Resources | 17,904.95 | 3,021.12 | 16,010.13 | 9,686.16 | 2,718.93 | 5,430.15 | **54,771.45** |
| Regulation and Maintenance | Gas Regulation | 128.06 | 9.53 | 102.88 | 65.19 | 16.04 | 9.14 | **330.85** |
|  | Climate Regulation | 21,130.29 | 2,238.20 | 18,436.20 | 11,618.93 | 3,022.26 | 3,358.99 | **59,804.86** |
|  | Disturbance | 670.39 | 79.55 | 630.28 | 356.55 | 120.78 | 779.18 | **2,636.72** |
|  | Water Regulation | 884.51 | 1,267.10 | 1,614.81 | 171.67 | 868.74 | 4,015.54 | **8,822.39** |
|  | Erosion Control | 3,589.99 | 464.46 | 3,219.70 | 1,971.50 | 546.82 | 1,328.19 | **11,120.66** |
|  | Soil Formation | 1,040.18 | 938.20 | 1,708.57 | 851.40 | 392.66 | 2,189.19 | **7,120.19** |
|  | Nutrient Cycling | 42.96 | 20.22 | 94.62 | 26.76 | 47.09 | 426.72 | **658.37** |
|  | Waste Treatment | 2,045.08 | 963.81 | 2,482.86 | 1,253.42 | 596.07 | 2,780.61 | **10,121.85** |
|  | Pollination | 363.35 | 62.46 | 320.28 | 192.42 | 54.39 | 111.66 | **1,104.54** |
|  | Biological Control | 209.14 | 78.15 | 283.26 | 132.23 | 108.07 | 390.26 | **1,201.11** |
|  | Habitat Refugia | 1,448.96 | 146.29 | 787.41 | 294.84 | 298.06 | 691.57 | **3,667.13** |
| Cultural Services | Recreation | 9,105.25 | 1,248.28 | 8,288.64 | 4,923.21 | 1,766.66 | 2,476.15 | **27,808.19** |
|  | Cultural | 162.43 | 34.61 | 88.44 | 9.40 | 25.28 | 489.33 | **809.50** |

**Table S2.** Value of the individual ecosystem service for each region in 2000

| **Million US/ha/year** | | **2000** | | | | | | |
| --- | --- | --- | --- | --- | --- | --- | --- | --- |
|  |  | **NMR** | **RRD** | **NCR** | **CHR** | **SER** | **MKD** | **Total** |
| Provisioning Services | Food | 7,239.99 | 4,283.01 | 8,672.75 | 5,117.04 | 1,592.38 | 9,659.25 | **36,564.42** |
|  | Material | 1,268.25 | 457.24 | 1,415.12 | 830.87 | 287.19 | 1,148.33 | **5,407.00** |
|  | Water | 1,264.57 | 1,022.54 | 1,835.67 | 910.80 | 512.25 | 2,496.30 | **8,042.14** |
|  | Genetic Resources | 17,568.29 | 2,947.35 | 15,851.59 | 9,383.63 | 2,824.73 | 5,258.20 | **53,833.79** |
| Regulation and Maintenance | Gas Regulation | 123.94 | 8.92 | 102.42 | 60.37 | 18.39 | 8.81 | **322.85** |
|  | Climate Regulation | 2,0561.27 | 2,217.86 | 18,378.34 | 10,932.44 | 3,349.36 | 3,292.07 | **58,731.34** |
|  | Disturbance | 649.04 | 113.22 | 649.97 | 330.64 | 125.92 | 1,204.79 | **3,073.57** |
|  | Water Regulation | 963.25 | 1,343.82 | 1,814.61 | 238.57 | 961.62 | 5,187.66 | **10,509.53** |
|  | Erosion Control | 3,501.21 | 486.17 | 3,220.46 | 1,868.60 | 585.76 | 1,673.20 | **11,335.41** |
|  | Soil Formation | 1,127.96 | 940.71 | 1,660.66 | 1,002.92 | 298.13 | 2,114.02 | **7,144.39** |
|  | Nutrient Cycling | 38.77 | 40.37 | 110.34 | 31.30 | 42.02 | 672.82 | **935.62** |
|  | Waste Treatment | 2,079.60 | 997.30 | 2,488.10 | 1,337.83 | 550.64 | 3,199.51 | **10,652.98** |
|  | Pollination | 356.55 | 60.59 | 316.72 | 186.83 | 56.27 | 108.07 | **1,085.03** |
|  | Biological Control | 202.97 | 88.17 | 295.33 | 151.61 | 98.51 | 522.98 | **1,359.57** |
|  | Habitat Refugia | 1,365.88 | 129.97 | 803.49 | 329.65 | 290.19 | 1,074.32 | **3,993.50** |
| Cultural Services | Recreation | 8,862.41 | 1,394.60 | 8,435.38 | 4,688.48 | 1,999.35 | 2,924.89 | **28,305.12** |
|  | Cultural | 155.28 | 51.97 | 97.59 | 8.93 | 20.96 | 779.02 | **1,113.75** |

**Table S3.** Value of the individual ecosystem service for each region in 2010

| **Million US/ha/year** | | **2010** | | | | | | |
| --- | --- | --- | --- | --- | --- | --- | --- | --- |
|  |  | **NMR** | **RRD** | **NCR** | **CHR** | **SER** | **MKD** | **Total** |
| Provisioning Services | Food | 7,618.42 | 3,954.91 | 8,128.46 | 5,324.61 | 1,198.09 | 9,332.92 | **35,557.42** |
|  | Material | 1,261.50 | 435.58 | 1,376.30 | 842.00 | 253.47 | 1,302.79 | **5,471.65** |
|  | Water | 1,300.30 | 982.65 | 1,754.04 | 987.70 | 425.83 | 2,347.60 | **7,798.11** |
|  | Genetic Resources | 17,298.41 | 2,849.61 | 15,807.93 | 9,164.60 | 2,914.57 | 4,808.76 | **52,843.89** |
| Regulation and Maintenance | Gas Regulation | 121.00 | 9.35 | 104.27 | 57.81 | 20.49 | 6.74 | **319.65** |
|  | Climate Regulation | 19,730.81 | 2,279.51 | 18,566.97 | 10,546.05 | 3,662.27 | 3,128.14 | **57,913.74** |
|  | Disturbance | 634.83 | 156.54 | 696.68 | 317.14 | 177.48 | 2,419.08 | **4,401.75** |
|  | Water Regulation | 1,006.28 | 1,468.47 | 1,945.04 | 382.26 | 1,002.67 | 6,573.12 | **12,377.83** |
|  | Erosion Control | 3,387.22 | 519.01 | 3,268.74 | 1,807.71 | 661.95 | 2,663.50 | **12,308.12** |
|  | Soil Formation | 1,158.74 | 863.64 | 1,519.13 | 1,054.53 | 205.57 | 1,993.35 | **6,794.96** |
|  | Nutrient Cycling | 44.51 | 63.86 | 133.23 | 34.45 | 56.52 | 1,373.12 | **1,705.68** |
|  | Waste Treatment | 2,089.59 | 990.71 | 2,445.44 | 1,375.15 | 526.68 | 4,214.18 | **11,641.75** |
|  | Pollination | 354.08 | 58.40 | 315.91 | 182.67 | 57.78 | 98.04 | **1,066.89** |
|  | Biological Control | 210.07 | 96.55 | 302.28 | 161.52 | 84.80 | 897.83 | **1,753.05** |
|  | Habitat Refugia | 1,662.66 | 158.59 | 891.26 | 355.25 | 247.13 | 2,046.93 | **5,361.82** |
| Cultural Services | Recreation | 8,574.66 | 1,685.42 | 8,807.84 | 4,625.57 | 2,233.77 | 3,798.52 | **29,725.79** |
|  | Cultural | 206.93 | 78.34 | 130.02 | 9.28 | 47.64 | 1,594.40 | **2,066.59** |

**Table S4.** Value of the individual ecosystem service for each region in 2020

| **Million US/ha/year** | | **2020** | | | | | | |
| --- | --- | --- | --- | --- | --- | --- | --- | --- |
|  |  | **NMR** | **RRD** | **NCR** | **CHR** | **SER** | **MKD** | **Total** |
| Provisioning Services | Food | 7,657.18 | 3,613.79 | 8,365.03 | 6,403.58 | 1,646.11 | 8,989.08 | **36,674.77** |
|  | Material | 1,252.37 | 436.49 | 1,423.94 | 920.70 | 291.33 | 1,377.58 | **5,702.41** |
|  | Water | 1,298.92 | 891.21 | 1,814.50 | 1,214.41 | 525.52 | 2,222.27 | **7,966.84** |
|  | Genetic Resources | 17,371.80 | 2,685.70 | 15,478.60 | 8,581.45 | 2,625.67 | 4,529.38 | **51,272.61** |
| Regulation and Maintenance | Gas Regulation | 122.47 | 9.32 | 100.80 | 49.05 | 16.47 | 5.96 | **304.08** |
|  | Climate Regulation | 19,516.73 | 2,302.53 | 18,147.71 | 9,296.56 | 3,119.22 | 3,135.79 | **55,518.55** |
|  | Disturbance | 702.42 | 357.44 | 835.11 | 279.69 | 206.91 | 3,090.72 | **5,472.29** |
|  | Water Regulation | 1,262.10 | 1,615.42 | 2,163.13 | 452.96 | 1,120.43 | 7,257.69 | **13,871.73** |
|  | Erosion Control | 3,416.22 | 677.59 | 3,322.12 | 1,624.21 | 616.71 | 3,212.58 | **12,869.44** |
|  | Soil Formation | 1,084.14 | 778.24 | 1,573.70 | 1,315.18 | 313.79 | 1,888.83 | **6,953.88** |
|  | Nutrient Cycling | 86.80 | 179.02 | 232.74 | 52.46 | 90.41 | 1,760.89 | **2,402.32** |
|  | Waste Treatment | 2,134.46 | 1,101.51 | 2,615.80 | 1,523.64 | 631.77 | 4,740.69 | **12,747.87** |
|  | Pollination | 358.60 | 54.78 | 309.29 | 171.89 | 52.31 | 91.75 | **1,038.62** |
|  | Biological Control | 237.22 | 154.86 | 378.39 | 209.18 | 118.03 | 1,106.31 | **2,203.99** |
|  | Habitat Refugia | 2,090.52 | 316.94 | 1,085.21 | 471.32 | 324.45 | 2,593.25 | **6,881.68** |
| Cultural Services | Recreation | 8,751.25 | 2,084.60 | 9,207.07 | 4,330.18 | 2,298.93 | 4,666.43 | **31,338.47** |
|  | Cultural | 307.37 | 211.30 | 231.44 | 16.31 | 81.18 | 2,043.20 | **2,890.81** |
